# Analysis of the Risk Factors for Short‐Term Outcomes in Acute Small Bowel Obstruction: A Retrospective Study

**DOI:** 10.1155/grp/8871353

**Published:** 2026-05-05

**Authors:** Yong Cai, Wei-Xuan Xu, Can-Hong Zhan, Qi-Hong Zhong, Shuai Chen, Hui Wang, Peng-Sheng Tu, Wen-Xuan Chen, Xian-Qiang Chen, Jun-Rong Zhang

**Affiliations:** ^1^ Department of General Surgery (Emergency Surgery), Fujian Medical University Union Hospital, Fuzhou, Fujian Province, China, fjmu.edu.cn

**Keywords:** acute small bowel obstruction, recurrence, risk factors, risk scoring models, short-term outcome

## Abstract

**Background:**

As the lack of analysis on risk factors for the short‐term outcomes of acute small bowel obstruction (ASBO), the current high readmission rate and severe postoperative complications of ASBO seriously affect the quality of life of patients. Risk‐scoring models for evaluating the short‐term outcomes of ASBO are still urgently needed.

**Methods:**

A total of 278 patients diagnosed with complete ASBO were included in this study. Cox proportional hazards regression analysis was used to construct predictive models for the length of stay (LOS) and the length of short‐term recurrence (LOR). The receiver operating characteristic (ROC) curves and the area under the curve (AUC) were calculated to assess the performance.

**Results:**

The mean LOS of 278 patients was 11.19 days, and 17 patients (6.1%) were readmitted. Multivariate analysis showed that longer duration of disease (Hazard ratio, HR = 1.551), low albumin (HR = 1.681), high lumen diameter(max) (HR = 1.477), low chlorine (HR = 1.046), and operation (HR = 7.456) were risk factors for LOS. Calcium (HR = 29.391) was an independent risk factor for LOR. Operation is not a risk factor for LOR. A risk‐scoring model of LOS (RS_LOS) was constructed to predict the posttreatment recovery (AUC, 30 days = 0.859). In the high‐risk group, the severe adverse events (SAE) rate reached 17.1%, exceeding 1.4% in the low‐risk group. The performance of RS_LOR was also satisfactory (AUC, 12 months = 0.802). In the high‐risk group, the recurrence rate reached 14.3%, nearly triple that in the low‐risk group (3.4%).

**Conclusion:**

The risk‐scoring models of LOS and LOR provide a comprehensive evaluation on the short‐term outcomes of ASBO treatment.

## 1. Introduction

Acute small bowel obstruction (ASBO), which commonly emerges in emergency status, accounts for 15%–20% of all acute general surgery admissions [1–3]. It causes a heavy burden on public health care, exceeding 2.3 billion in costs annually since 2005, and its cost is continuing to rise [4]. In the United States, approximately 3.5% of all hospital admissions and 16% of all surgical admissions are diagnosed as ASBO [5, 6]. ASBO is a closely time‐related disease; the progression, the severity, the in‐hospital recovery, and the recurrence all evolve with time [7, 8]. Lack of comprehensive analysis on risk factors regarding time‐related outcomes including length of stay (LOS) and length of short‐term recurrence (LOR) would cause repeated hospitalization, which hinders the effective management of ASBO [5, 9].

Several previous reports unraveled risk factors of adverse outcomes for ASBO. Old age, severe peritonitis, lengthened operation time, high white blood cell (WBC), and procalcitonin (PCT) are closely correlated to severe ASBO [10]. Chronic nephropathy, obstructive pulmonary disease, and perioperative blood transfusion independently increased the morbidity and mortality of ASBO [11]. For LOR, the selection between conservative treatment and surgical intervention is still in debate. Even though early operation would reduce nearly 50% recurrence rates, the formation of new adhesions also poses a challenge to surgeons [12]. Moreover, successful conservative treatment may also leave adhesions, inducing future episodes of bowel obstruction with a high frequency of recurrence [13].

In this study, LOS and LOR were applied to assess the short‐term outcomes for in‐hospital and out‐hospital recovery of ASBO [14–18]. Cox regression was used to identify the independent risk factors and construct nomogram risk scoring models on LOS and LOR for ASBO.

## 2. Methods

### 2.1. Population

From October 2016 to February 2021, 479 patients diagnosed with intestinal obstruction at Fujian Medical University Union Hospital were included in the study. For clinical practice, a preliminary diagnosis of small bowel obstruction can be established according to both clinical findings such as the crampy abdominal pain, distention, vomiting, retention of stool and flatus, and the high‐pitched or absent bowel sounds; and radiographic findings such as the small bowel dilated greater than 2.5 cm without colon dilation, the obstructive point, and the intraluminal air–fluid levels. A total of 278 patients were included (the workflow is shown in Figure S1). The inclusion criteria were as follows: (1) symptoms and (2) radiological evidence confirmed completed small bowel obstruction. The exclusion criteria were as follows: (1) large intestine obstruction, (2) CT deletion, and (3) incomplete clinical data. All these patients were followed up for a total of 1 year.

This study was performed in accordance with relevant principles outlined in the Declaration of Helsinki and it was approved by Fujian Medical University Union Hospital Ethics Committee. Written informed consent was obtained from all enrolled patients.

### 2.2. Clinical Variable Definition

Baseline characteristics, including clinical signs and symptoms, laboratory examination and radiological features of CT, were entered into the database (shown in Table 1). Categorical variables, especially the disease duration, were converted from continuous variables according to median level of the patient′s pain duration [19]. Abdominal pain, abdominal distention, vomiting, retention of stool and flatus, tenderness, rebound tenderness, and bowel sounds are clinical features [20–22]. Combinations of inflammatory parameters, such as the neutrophil‐to‐lymphocyte ratio (NLR) and lymphocyte‐to‐monocyte ratio (LMR), were calculated and recorded accordingly. The CT features recorded in this study were included: mesenteric fluid, ascites, spiral sign, concentric circle sign, small bowel fecal sign, edema of bowel wall, bowel wall thickness, and luminal diameter_(max)_ [19–27]. The bowel wall thickness and luminal diameter_(max)_ levels were classified by the median. All CT scan images were cross‐reviewed and evaluated by two senior general surgeons with extensive experience in emergency abdominal surgery and two radiologists.

**Table 1:** Univariate analysis of cox proportional risk regression.

Characteristics	LOS^a^		LOR^b^	
	Hazard ratio (95% CI)	p value	Hazard ratio (95% CI)	p value
Gender (female)	1.087 (0.844–1.401)	0.518	7.771 (1.031–58.60)	**0.047**
Age (year)	0.994 (0.986–1.002)	0.151	1.013 (0.980–1.047)	0.450
Weight (kg)	1.012 (1.002–1.023)	**0.020**	1.000 (0.960–1.041)	0.982
BMI (kg/m^2^)		0.184		0.358
18.5–23.9	Reference		Reference	
≤ 18.5	1.290 (0.900–1.850)		1.470 (0.380–5.700)	
> 23.9	0.780 (0.570–1.060)		1.690 (0.540–5.310)	
Comorbidity (none)	0.929 (0.707–1.221)	0.599	2.192 (0.834–5.758)	0.111
Temperature (°C)	1.058 (0.807–1.387)	0.682	0.727 (0.205–2.578)	0.621
Length of disease (> 3 days)	1.539 (1.206–1.965)	**< 0.001**	1.627 (0.573–4.617)	0.361
Treatment (operation)	4.432 (3.349–5.866)	**< 0.001**	0.773 (0.294–2.032)	0.602
Abdominal pain		0.983		0.829
None or mild	Reference		Reference	
Moderate	0.930 (0.650–1.340)		1.100 (0.240–5.030)	
Severe	0.970 (0.650–1.450)		1.190 (0.230–6.140)	
Abdominal distention (none)	0.989 (0.758–1.290)	0.936	2.851 (0.652–12.47)	0.164
Vomiting (none)	0.893 (0.692–1.152)	0.385	1.510 (0.492–4.630)	0.471
Retention of stool and flatus (none)	0.966 (0.758–1.232)	0.782	0.894 (0.340–2.348)	0.819
Tenderness (none)	1.184 (0.828–1.692)	0.354	0.458 (0.149–1.403)	0.172
Rebound tenderness (none)	0.852 (0.647–1.124)	0.258	0.662 (0.190–2.302)	0.516
Bowel sounds		0.261		0.125
Normal	Reference		Reference	
None or low	1.020 (0.780–1.320)		1.330 (0.410–4.370)	
High or hyperactive	1.230 (0.890–1.700)		2.580 (0.790–8.440)	
History of abdominal operation (none)	0.960 (0.734–1.257)	0.769	0.631 (0.234–1.707)	0.365
Adhesive intestinal obstruction (none)	1.050 (0.825–1.337)	0.692	1.270 (0.470–3.433)	0.638
Neoplastic intestinal obstruction (none)	0.558 (0.319–0.976)	**0.041**	1.262 (0.167–9.516)	0.821
WBC (≤10.15 × 10^9^/L)	0.943 (0.718–1.238)	0.672	1.232 (0.434–3.497)	0.695
NE% (≤ 80.40)	1.001 (0.761–1.316)	0.996	0.942 (0.307–2.890)	0.917
NLR (≤ 2)	0.921 (0.613–1.384)	0.694	1.672 (0.222–12.61)	0.618
LMR (≤ 2.05)	1.430 (1.127–1.815)	**0.003**	0.677 (0.258–1.778)	0.428
Lymphocyte (10^9^/L)	1.299 (1.041–1.621)	**0.021**	0.562 (0.200–1.583)	0.275
Hb (g/L)	1.009 (1.003–1.015)	**0.003**	1.013 (0.989–1.038)	0.288
PLT (10^9^/L)		**0.032**		0.780
100–300	Reference		Reference	
< 100	0.800 (0.330–1.940)		3.690 (0.480–28.210)	
> 300	0.710 (0.510–0.970)		1.090 (0.310–3.830)	
Albumin (≤ 40g/L)	1.347 (1.012–1.792)	**0.041**	1.509 (0.558–4.079)	0.418
Sodium (mmol/L)	1.026 (0.517–0.939)	0.064	0.933 (0.831–1.048)	0.243
Potassium (mmol/L)	0.954 (0.785–1.160)	0.640	2.525 (1.239–5.148)	**0.011**
Calcium (mmol/L)	1.718 (0.951–3.103)	0.073	18.96 (1.888–190.5)	**0.012**
Chlorine (mmol/L)	1.028 (1.002–1.055)	**0.032**	0.938 (0.854–1.030)	0.178
Glucose (mmol/L)	0.971 (0.937–1.006)	0.104	0.933 (0.783–1.112)	0.438
Spiral signs (none)	0.774 (0.558–1.072)	0.124	2.363 (0.832–6.707)	0.106
Mesenteric fluid		**0.020**		0.922
None	Reference		Reference	
Local	0.980 (0.670–1.430)		0.340 (0.060–1.860)	
Diffuse	0.730 (0.520–1.010)		0.760 (0.240–2.390)	
Concentric circle sign (none)	0.629 (0.367–1.078)	0.092	0.000 (0.000–Inf)	0.997
Edema of bowel wall (none)	0.661 (0.514–0.851)	**0.001**	1.199 (0.422–3.404)	0.733
Ascites (none)	0.732 (0.565–0.948)	**0.018**	1.037 (0.365–2.943)	0.946
Small bowel feces sign (none)	1.293 (1.020–1.640)	**0.037**	1.045 (0.403–2.708)	0.928
Bowel wall thickness (≤ 3.36 mm)	0.734 (0.579–0.930)	**0.010**	0.691 (0.263–1.814)	0.452
Luminal diameter_(max)_ (> 4.285 cm)	1.366 (1.074–1.737)	**0.011**	0.899 (0.347–2.331)	0.827
Strangulated bowel obstruction (none)	0.469 (0.339–0.650)	**< 0.001**	0.703 (0.161–3.075)	0.640
Degree of mucosal ischemia		**< 0.001**		0.671
None	Reference		Reference	
Ischemia	0.530 (0.350–0.820)		0.680 (0.090–5.150)	
Necrosis	0.410 (0.260–0.650)		0.730 (0.100–5.510)	
Intraoperative blood loss (mL)	0.996 (0.994–0.999)	**0.002**	0.994 (0.974–1.013)	0.525
Bowel resection (none)	0.345 (0.253–0.471)	**< 0.001**	0.575 (0.132–2.515)	0.462

*Note:* Values marked with “^a^” were presented as mean. Values marked with “^b^” were binary variables bounded by the median. Bolded data are used to indicate category subheadings for better readability.

Abbreviation: NA, not accessible

Treatment details were collected and recorded from the discharge summary, including treatment type (conservation or operation), the feature of the obstruction, the amount of blood loss, bowel resection, and postoperative pathology.

### 2.3. Outcome Definition

LOS was defined as the interval from the first admission to discharge. Readmission was defined as a secondary admission due to recurrence of ASBO within 12 months. LOR was defined as the interval between the first discharge and the second readmission. The severity of complications was graded according to the Clavien–Dindo (CD) system [28, 29], CD Grade IV–Grade V were classified as SAE.

### 2.4. Statistical Analysis

Cox proportional hazards regression is commonly used to analyze time‐related records [30]. In this study, Cox regression was used to analyze LOS, in which the terminal event was first discharge, and LOR, in which the terminal event was second readmission due to recurrence of ASBO. That is, for LOS, a hazard ratio (HR > 1) indicates a protective factor, meaning that increasing scores are associated with reduced LOS, and for LOR, the factor (HR > 1) is a risk factor (increasing scores indicate accelerating probability of readmission and reducing LOR). Multivariate Cox regression analysis was used to define the independent risk factors (*p* < 0.05). Kaplan–Meier plotting was used to assess the relationship between LOS and each risk factor. For LOS, we extracted the following risk scoring formulas: risk score (RS_LOS) = [0.632×(disease duration)+1.785×(treatment)+0.466×(albumin)+0.026×(chlorine)‐0.364×(luminal diameter(max))]. Receiver operating characteristic (ROC) curves and the area under the curve (AUC) were calculated to assess the accuracy of the model. Akaike′s information criterion (AIC) and AUC were simultaneously used to evaluate the performance and efficacy of the predictive model. We also determined the RS__LOR_ = [1.892 × (gender) + 0.832 × (potassium) + 2.785 × (calcium)]. All of the statistical analyses were performed in R Version 4.1.3. If the *p* value was lower than 0.05, the parameter was considered statistically significant.

## 3. Results

### 3.1. Baseline and Surgical Outcomes of ASBO

All 278 patients were included in this study, and their characteristics are shown in Table 2. Of these patients, 17 presented with recurrent bowel obstruction, and their days to recurrence, first diagnosis, and treatment were detailed in Table S1. Nearly 57 (25%) had inadequate nutrition support (body mass index, BMI ≤ 18.5 kg/m^2^). Meanwhile, 165 (59.6%) patients had a disease duration of less than 3 days, and 180 (64.7%) patients underwent conservative treatment. A total of 206 (74.1%) patients had a history of previous surgery, and the majority of intestinal obstruction was adhesive in 165 patients (59.4%); other etiology of intestinal obstruction was also presented in Table S1. For laboratory tests, the median Hb was 128 g/L, and the median electrolytes included sodium 138.1 mmol/L, potassium 4.02 mmol/L, chloride 102 mmol/L, and calcium 2.17 mmol/L. For radiological features, 185 (66.5%) patients were confirmed to have intestinal wall edema, 194 (69.8%) patients had ascites, and 144 (51.8%) patients had small bowel feces signs. The median diameter of the largest distended bowel for each case was 4.285 cm.

**Table 2:** Patient characteristics.

Characteristics	Total (n=278)	Characteristics	Total (n=278)
Gender, n (%)		Neoplastic intestinal obstruction, n (%)	
Male	189 (68.0%)	None	265 (95.3%)
Female	89 (32.0%)	Yes	13 (4.7%)
Age (years) ^∗^	62 (50–70)	**Symptoms and physical signs**	
Weight (kg) ^∗^	55 (49.75–62.50)	Abdominal pain, n (%)	
BMI, n (%)		None or mild	36 (13.0%)
18.5–23.9	132 (57.9%)	Moderate	165 (59.6%)
≤18.5	57 (25.0%)	Severe	76 (27.4%)
>23.9	39 (17.1%)	Abdominal distention, n (%)	
Comorbidity, n (%)		None	76 (27.3%)
none	209 (75.2%)	Yes	202 (72.7%)
yes	69 (24.8%)	Vomiting, n (%)	
Temperature (°C) ^∗^	36.6 (36.5–36.8)	None	87 (31.3%)
Length of disease, (%)		Yes	191 (68.7%)
≤3 days	165 (59.6%)	Retention of stool and flatus, n (%)	
>3 days	112 (40.4%)	None	107 (38.5%)
Treatment, n (%)		Yes	171 (61.5%)
Conservative	180 (64.7%)	Yes	243 (87.4%)
Operation	98 (35.3%)	Rebound tenderness, n (%)	
History of abdominal operation, n (%)		None	211 (75.9%)
None	72 (25.9%)	Yes	67 (24.1%)
Yes	206 (74.1%)	Bowel sounds, n (%)	
Adhesive intestinal obstruction, n (%)		Normal	117 (42.1%)
None	113 (40.6%)	None or low	105 (37.8%)
Yes	165 (59.4%)	High or hyperactive	56 (20.1%)
Laboratory indicators	Laboratory indicators	Glucose (mmol/L) ^∗^	7.15 (5.58‐9.20)
WBC (10^9^/L), n (%)		**CT imaging features**	
≤10.15	208 (74.8%)	Spiral signs, n (%)	
>10.15	70 (25.2%)	None	235 (84.5%)
NE%, n (%)		Yes	43 (15.5%)
≤80.40	210 (75.5%)	Concentric circle sign, n (%)	
>80.40	68 (24.5%)	None	264 (95.0%)
NLR, n (%)		Yes	14 (5.0%)
≤2	252 (89.7%)	Mesenteric fluid, n (%)	
>2	26 (10.3%)	None	46 (16.5%)
LMR, n (%)		Local	66 (23.7%)
≤2.05	137 (49.3%)	Diffuse	166 (59.8%)
>2.05	141 (50.7%)	Edema of bowel wall, n (%)	
Lymphocyte (10^9^/L) ^∗^	0.97 (0.70–1.37)	None	93 (33.5%)
Hb (g/L) ^∗^	128.00 (114.00–140.75)	Yes	185 (66.5%)
PLT, n (%)		Ascites, n (%)	
100–300	224 (81.2%)	None	84 (30.2%)
<100	5 (1.8%)	Yes	194 (69.8%)
>300	47 (17.0%)	Small bowel feces sign, n (%)	
Albumin, n (%)		None	134 (48.2%)
≤40	181 (73.6%)	Yes	144 (51.8%)
>40	65 (26.4%)	Bowel wall thickness, n (%) ^∗∗∗^	3.36
Sodium (mmol/L) ^∗^	138.1 (136.0–140.6)	Luminal diameter_(max)_, n (%) ^∗∗∗^	4.285
Potassium (mmol/L) ^∗^	4.02 (3.75–4.37)		
Chlorine (mmol/L) ^∗^	102.0 (99.0–104.1)		
Calcium (mmol/L) ^∗^	2.17 (2.03–2.32)		
Pathological and intraoperative conditions	Pathological and intraoperative conditions	Etiology	
Strangulated bowel obstruction, n (%)		Adhesion	165 (59.4%)
None	234 (84.2%)	Volvulus	25 (9.0%)
Yes	44 (15.8%)	Intussusception	7 (2.5%)

Degree of mucosal ischemia, n (%)		Intestinal bezoar	13 (4.7%)
None	234 (84.2%)	Tumor	13 (4.7%)
Ischemia	23 (8.3%)	Inflammatory bowel disease	10 (3.6%)
Necrosis	21 (7.5%)	Anastomotic stenosis	1 (0.4%)

Intraoperative blood loss (mL) ^∗∗^	18.76±81.90 mL	Hernia	18 (6.5%)
Bowel resection, n (%)		Ischemia bowel disease	5 (1.8%)
None	226 (81.3%)	Other diseases	14 (5.0%)
Yes	52 (18.7%)	NA	7 (2.5%)

*Note:* Values marked with “ ^∗^” were presented as median (interquartile range).Values marked with “ ^∗∗^” were presented as mean. Values marked with “ ^∗∗∗^” were binary variables bounded by the median.

Abbreviations: BMI, body mass index; WBC, white blood cell; NE%, neutrophil percentage; NLR, neutrophil to lymphocyte ratio; LMR, lymphocyte to monocyte ratio; Hb, hemoglobin; PLT, platelet; NA, not accessible.

### 3.2. Univariate Analysis of LOS

The mean LOS for ASBO was 11.19 days. Via univariate Cox analysis, lower body weight (*p* = 0.020), prolonged disease duration (*p* ≤ 0.001), neoplastic intestinal obstruction (*p* = 0.041), and operation (*p* ≤ 0.001) were closely related to longer LOS. Similarly, a lower LMR (*p* = 0.003) and lower levels of lymphocytes (*p* = 0.021), Hb (*p* = 0.003), albumin (*p* = 0.041), abnormal PLT (*p* = 0.032), and chlorine (*p* = 0.032) were closely correlated with a longer LOS. For radiomic features, the occurrence of mesenteric fluid (*p* = 0.020), edema of the bowel wall (*p* = 0.001), ascites (*p* = 0.018), thickened bowel wall (*p* = 0.010), distended luminal diameter (*p* = 0.011) and lack of fecal signs (*p* = 0.037) were also closely related to extended LOS (Table 1 and Figure S2a).

### 3.3. Multivariate Analysis of LOS

When using Cox regression multivariate analysis of LOS, the factor (HR > 1) was a protective factor. In the end, a shorter disease duration (HR = 1.551, *p* = 0.014), higher level of albumin (HR = 1.681, *p* = 0.007), normal chlorine (HR = 1.046, *p* = 0.012), smaller luminal diameter_(max)_ (HR = 1.477, *p* = 0.015), and conservative treatment (HR = 7.456, *p* ≤ 0.001) were independent protective factors for LOS (Table 3). Decreasing levels of chloride increase the risk of prolonged LOS. Kaplan–Meier plotting showed that over 3 days of symptoms (*p* ≤ 0.001) (Figure 1a), lower ALB (< 40 g/L, *p* = 0.039) (Figure 1b), more extended luminal diameter_(max)_ (> 4.285 cm, *p* = 0.01) (Figure 1d), and operation (*p* ≤ 0.001) (Figure 1c) were significantly associated with prolonged LOS.

**Table 3:** Multivariate analysis of Cox proportional risk regression.

Characteristics	LOS^a^		LOR^b^	
	Hazard Ratio (95% CI)	p value	Hazard ratio (95% CI)	p value
Gender Male versus female			6.375 (0.843–48.18)	0.073
Length of disease ≤ 3 days versus > 3 days	1.551 (1.094–2.198)	**0.014**		
Treatment Conservative versus operation	7.456 (4.441–12.517)	**< 0.001**		
Albumin > 40 g/L versus ≤ 40 g/L	1.681 (1.155–2.449)	**0.007**		
Potassium (mmol/L)			2.180 (0.998–4.760)	0.051
Calcium (mmol/L)			29.391 (1.810–302.235)	**0.016**
Chlorine (mmol/L)	1.046 (1.010–1.083)	**0.012**		
Luminal diameter_(max)_ ≤ 4.285 versus > 4.285 cm	1.477 (1.080–2.020)	**0.015**		

*Note:* Bold values indicate statistical significance (*p* < 0.05).

Abbreviations: CI, confidence interval; LOR, length of readmission; LOS, length of stay.

^a^Multivariate analysis of short‐term prognosis.

^b^Multivariate analysis of long‐term prognosis.

Figure 1Kaplan–Meier survival curve of independent risk factor. This figure was based on Kaplan–Meier plotting to assess the relationship between LOS and each independent prognostic factor. (a) Kaplan–Meier plotting of disease duration, (b) Kaplan–Meier plotting of albumin, (c) Kaplan–Meier plotting of treatment, and (d) Kaplan–Meier plotting of luminal diameter (max).

**Figure:**
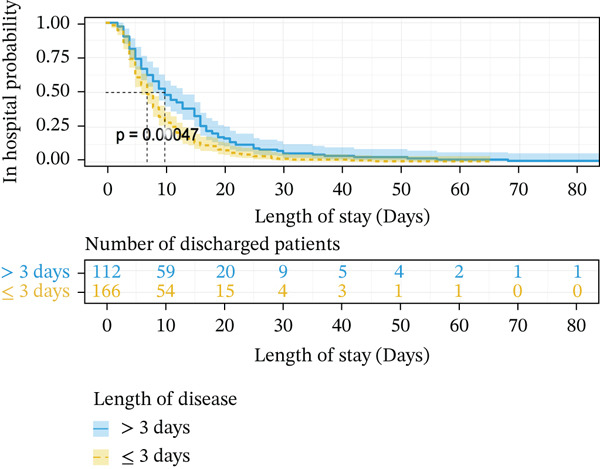
(a)

**Figure:**
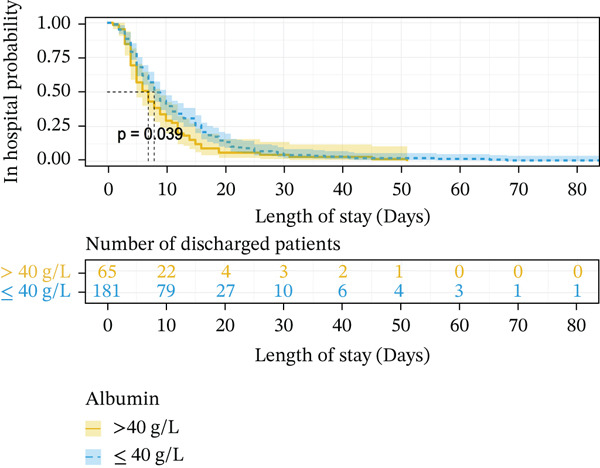
(b)

**Figure:**
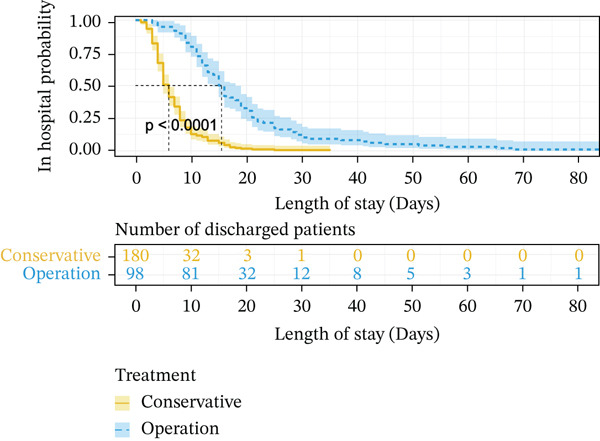
(c)

**Figure:**
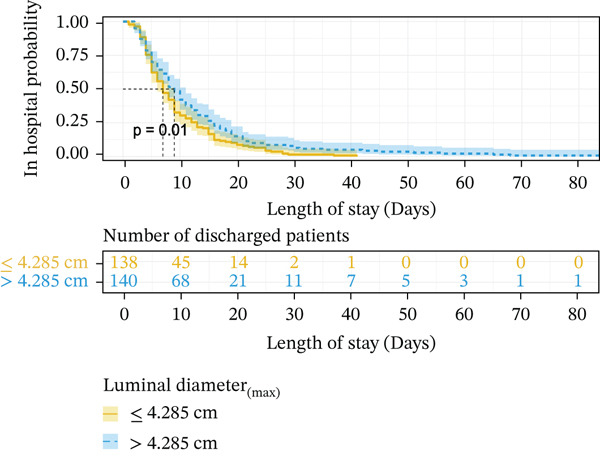
(d)

### 3.4. Univariate and Multivariate Analyses of LOR

When using Cox regression multivariate analysis of LOR, the factor (HR > 1) was a risk factor. Of all the patients, 17 (6.1%) were readmitted to the hospital due to the recurrence of ASBO. Males (*p* = 0.047) were more likely to have recurrent obstruction. LOR was also significantly associated with electrolyte imbalance, including high levels of potassium (*p* = 0.011) and calcium (*p* = 0.012) at first admission (Table 1 and Figure S2b). The Cox proportional hazards regression model confirmed that only the accumulating level of calcium was an independent risk factor for LOR (HR = 29.391, 95% CI 1.810–302.235, *p* = 0.016) (Table 3).

### 3.5. Construction of the Risk Scoring Model for LOS

Based on the regression coefficient for each factor, we constructed a multidimensional model termed the RS__LOS_ score. The AUC of the RS__LOS_ score for patients within 10 days of LOS, 20 days of LOS, and 30 days of LOS was 0.929, 0.883, and 0.883, respectively (Figure 2a). A nomogram was also drawn to directly calculate the probability of the occurrence of discharge (Figure 3a), patients with a symptom length of over 3 days (*p* ≤ 0.001), lower ALB (< 40 g/L, *p* = 0.039), more extended luminal diameter_(max)_ (> 4.285 cm, *p* = 0.01), and operation (*p* ≤ 0.001) would have longer LOS. Compared with the low‐risk group (score = 0), the high‐risk group (score = 1) was more likely to have SAE (*p* ≤ 0.001) (Table S2).

Figure 2Time‐related ROC curve for LOS and LOR. This figure shows the prediction performance of LOS and LOR, measured by the receiver operating characteristic curve (ROC) and displayed by the area under the ROC curve (AUC). (a) The ROC curve and AUC of LOS and (b) The ROC curve and AUC of LOR.

**Figure:**
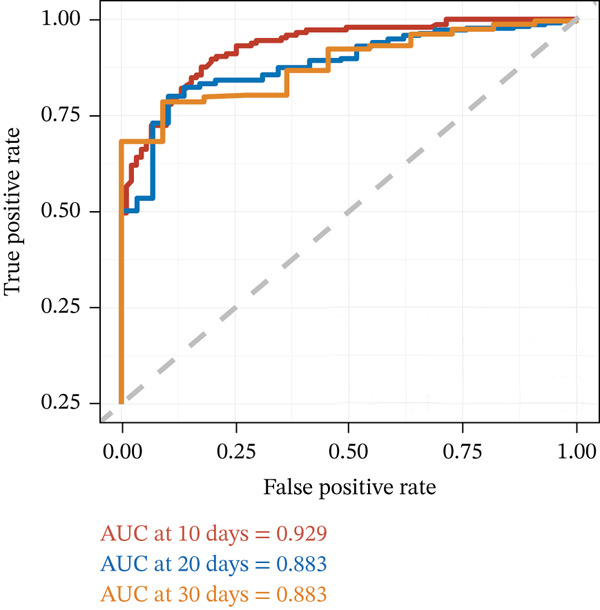
(a)

**Figure:**
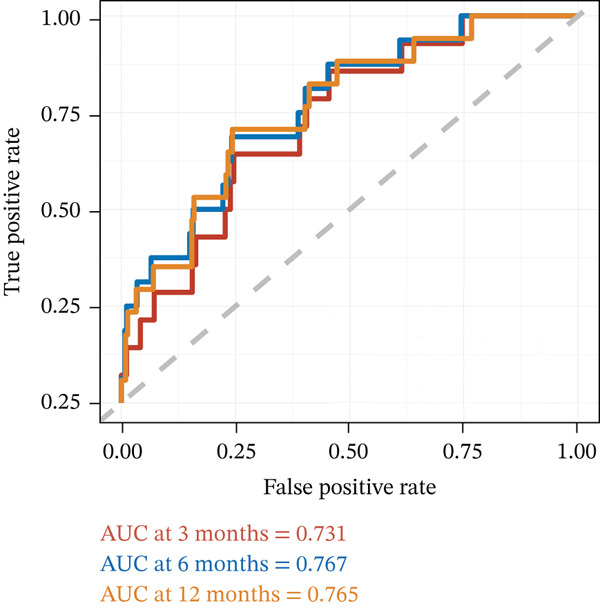
(b)

Figure 3Nomogram for LOS and LOR. Treatment: 1‐conservative, 0‐operation; disease duration: 1–≤ 3 days, 0–> 3 days; albumin: 1–> 40 g/L, 0–≤ 40 g/L; luminal diameter(max): 1–≤ 4.285 cm, 0–> 4.285 cm; gender: 1‐male, 0‐female. (a) Nomogram for the risk scoring model of LOS and (b) nomogram for the risk scoring model of LOR.

**Figure:**
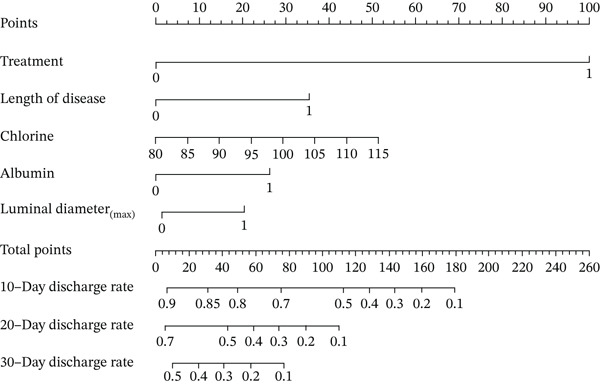
(a)

**Figure:**
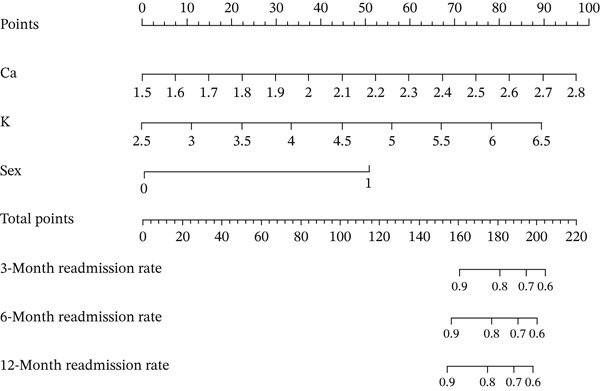
(b)

### 3.6. Construction of the Risk Scoring Model for LOR

The RS__LOR_ model included calcium, potassium, and sex showed the highest accuracy (3 months, AUC = 0.731; 6 months, AUC = 0.767; 12 months, AUC = 0.765) and the lowest AIC score (179.53) (Table 4, Figure 2b). Compared with the low‐risk group (score = 0), the high‐risk group (score = 1) was more likely to have readmissions (*p* = 0.003, RR = 4.206) (Table 5). A nomogram was drawn to directly calculate the probability of the recurrence (Figure 3b).

**Table 4:** Comparison of different predictive models for LOR.

Regression model	Correlation factor		AIC	AUC: 3 months	AUC: 6 months	AUC: 12 months
	Gender	Potassium				
Calcium	−	−	185.94	0.628	0.675	0.687
	−	+	183.47	0.680	0.721	0.725
	+	−	181	0.704	0.743	0.744
	+	+	179.53	0.731	0.767	0.765

Abbreviations: AIC, Akaike′s information criterion. AUC, area under the curve; LOR, length of readmission.

**Table 5:** The discriminative effectiveness of LOR score.

	Non‐readmission (*n* = 261)	Readmission (*n* = 17)	p value
Score		0.003	
0, *n* (%)	201 (96.6%)	7 (3.4%)	1
Conservative	130 (96.3%)	5 (3.7%)	
Operation	71 (97.3%)	2 (2.7%)	
1, *n* (%)	60 (85.7%)	10 (14.3%)	0.508
Conservative	40 (88.9%)	5 (11.1%)	

Abbreviation: LOR, length of readmission.

## 4. Discussion

Currently, with the hospitalization costs ranging from $29,000 to $86,000 per admission and $9–11.4 billion per year in the United States, ASBO remains a substantial burden on health care and financial resources [4, 31]. Owing to the complicated etiology of ASBO and inappropriate management at first admission, recurrent episodes that significantly deteriorate patients′ quality of life [32]. Regarding the less attention to the evaluation of short‐term outcomes, the length of stay (LOS) and length of recurrence (LOR) were introduced to evaluate both in‐hospital and postcharge recovery of ASBO. According to independent risk factors and predictive models, early interventions might improve short‐term outcomes for ASBOs.

For LOS, longer disease duration, low albumin, high lumen diameter_(max)_, low chlorine, as well as operation were observed as independent risk factors. As intestinal obstruction developed, prolonged duration days exacerbated the obstruction degree. The bowel lumen extremely dilated with accumulating air and intestinal fluid, leading to the elevating intramural tension [34, 37]. This progression aggravated bowel wall edema and microvascular damage, consequently as the worse bowel function and delayed recovery [38–41]. Accordingly, both the disease duration and maximal lumen diameter_(max)_ were defined as independent risk factors for longer LOS in our study. Moreover, the secondary malfunction in reabsorption of water and electrolytes caused significant disbalance in water–electrolyte homeostasis, as hypochloremia, as closely associated with metabolic alkalosis of ASBO [42, 43], was also defined as a risk factor for LOS. Owing to the insufficient energy support and dysfunction in intestinal physiology, hypoalbuminemia was another risk factor for LOS in ASBO [34, 36]. Furthermore, with a high coefficient, operation was observed as another independent risk factor for a longer LOS, which may correlate with severe postoperative complications, ICU admission, and mortality [8, 44, 45]. Even so, inappropriate conservative management of ASBO would increase the recurrence rates with a shorter LOR than surgical counterparts [1, 12, 44]. The choice between operation and conservation needs further analysis of risk factors regarding LOR. A risk scoring model for the evaluation on LOS was constructed. Demonstrating robust discriminative capacity, it also well distinguished SAE patients from the non‐SAE between low‐risk (1.4%) and high‐risk (17.1%) group.

In contrast to the factors affecting LOS, the three independent risk factors for LOR were hypercalcemia, hyperkalemia, and male sex. For ASBO, with the deteriorating bowel vitality, intestinal ischemia and hypoxia gradually destroy the epithelial barrier. Subsequent influx of calcium and potassium into cells resulted in calcium and potassium overload, which consequently initiated programmed cell death of the intestinal epithelium [46, 47], leading to the hypercalcemia and hyperkalemia as independent risk factors for LOR. Among the 17 patients with recurrent readmissions in this study, only one was female, suggesting male sex was another risk factor for LOR. This observation may owe to estrogen‐mediated reperfusion after intestinal ischemia, which could reduce the production of H_2_O_2_[48]. In previous studies, early adhesiolysis during the first episode of adhesive SBO was associated with a lower recurrence rate [13]. Remarkably, different from LOS, our findings indicated the types of treatment would not significantly affect LOR. In another word, rather than fostering new adhesion, early surgical intervention at first admission might only extend LOS but not LOR.

Finally, based on the previous three components, a risk scoring model for LOR was constructed, yielding an AUC of 0.731 for 3 months, 0.767 for 6 months, and 0.765 for 12 months. According to risk patient stratification, the recurrence rate was only 3.4% in the low‐risk group. However, in the high‐risk group, the recurrence rate reached 14.3%, nearly triple that in the low‐risk group, indicating an excellent differentiating ability of the predictive model in our study.

Several limitations exist in this study. First, this study was a retrospective study conducted in a single center. Second, some parameters may not be identifiable due to the small sample size and short follow‐up period. Third, these risk models should be validated in other centers in the future to support their efficacy.

## 5. Conclusion

The risk scoring models of LOS and LOR provide a comprehensive evaluation of the short‐term outcomes of ASBO treatment. Operation is not an independent risk factor for LOR. According to the risk scoring model, early targeted intervention should be adjusted accordingly.

## Author Contributions

Yong Cai designed and performed the research and drafted the manuscript; Jun‐Rong Zhang designed the research and supervised and reviewed the report; Xian‐Qiang Chen supervised the report and provided funding acquisition; Wei‐Xuan Xu, Can‐Hong Zhan, and Qi‐Hong Zhong designed the research and contributed to the analysis; Shuai Chen, Hui Wang, Peng‐Sheng Tu, and Wen‐Xuan Chen collected data and provided methodology. Yong Cai and Wei‐Xuan Xu have contributed equally to this work.

## Funding

This study was supported by the Construction Project of Fujian Province Minimally Invasive Medical Center ([2021]76).

## Conflicts of Interest

The authors declare no conflicts of interest.

## Supporting Information

Additional supporting information can be found online in the Supporting Information section.

## Supporting information


**Supporting Information 1** Figure S1: Workflow of this study.

**Supporting Information 2** Figure S2: Subgroup forest map of LOS and LOR univariate analysis. This figure showed risk factors for LOS and LOR. (a) The subgroup forest map of LOS and (b) the subgroup forest map of LOR.

**Supporting Information 3** Table S1: The diagnosis and treatment of patients with recurrent acute small bowel obstruction at first. Table S2: The discriminative effects of the risk scoring model on LOS.

## Data Availability

The data will be available on request from the authors.
