# Supplementary figures and images for: Analysis of the Risk Factors for Short‐Term Outcomes in Acute Small Bowel Obstruction: A Retrospective Study
Source: Gastroenterol Res Pract. 2026 May 5;2026:8871353. doi: 10.1155/grp/8871353 (PMC13140370; doi:10.1155/grp/8871353)

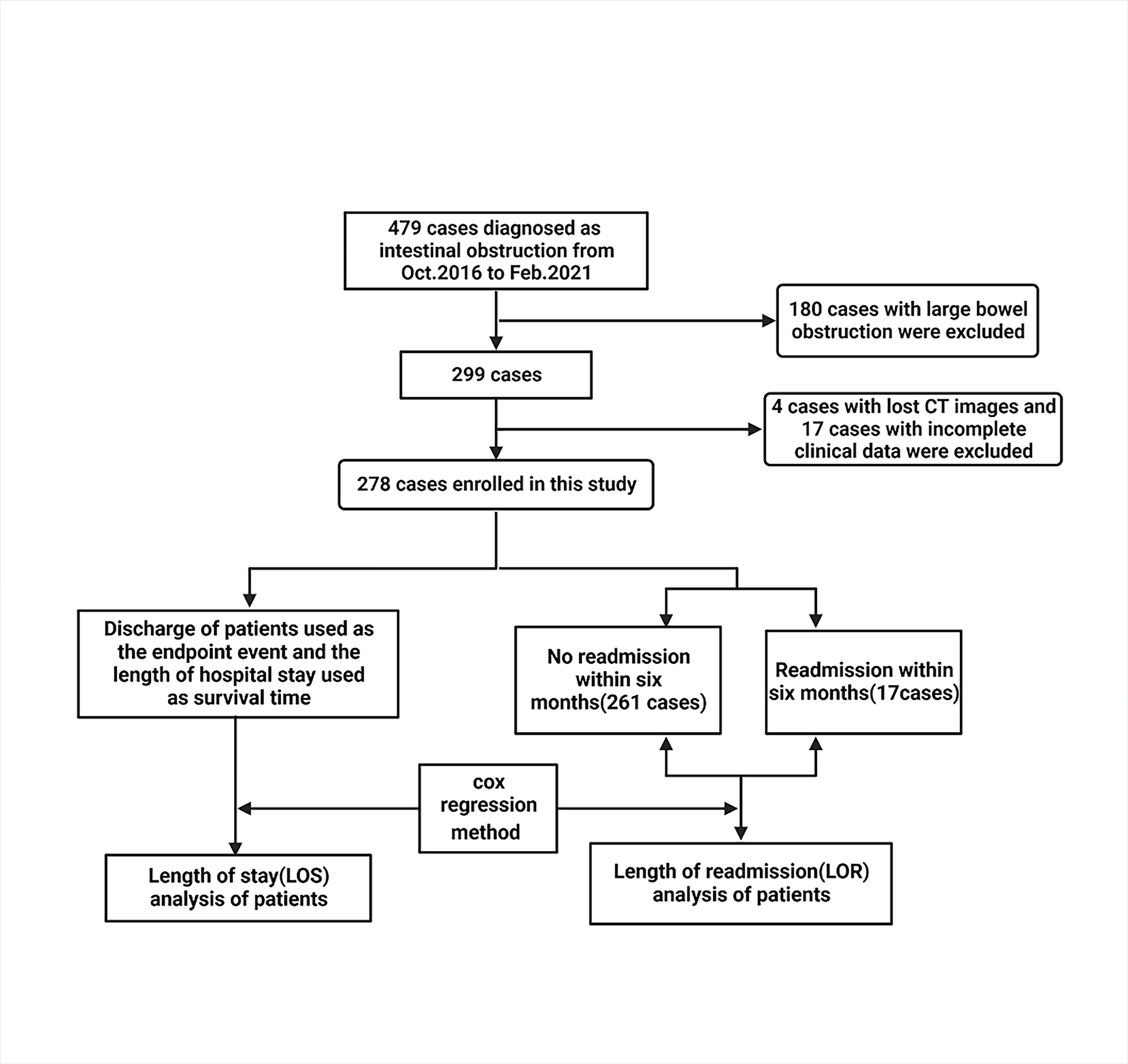

Supplement: Supplementary file 1 — Supporting Information 1 Figure S1: Workflow of this study. [file GRP-2026-8871353-s001.tiff]
