# Supplementary material for: Analysis of the Risk Factors for Short‐Term Outcomes in Acute Small Bowel Obstruction: A Retrospective Study
Source: Gastroenterol Res Pract. 2026 May 5;2026:8871353. doi: 10.1155/grp/8871353 (PMC13140370; doi:10.1155/grp/8871353)
Supplement: Supplementary file 3 — Supporting Information 3 Table S1: The diagnosis and treatment of patients with recurrent acute small bowel obstruction at first. Table S2: The discriminative effects of the risk scoring model on LOS. [file GRP-2026-8871353-s003.docx]

**Supplementary material**

**Supplementary table 1: The diagnosis and treatment of patients with recurrent acute small bowel obstruction at first**

| Names of patients  (Recurrence time/day) | First diagnosis | Treatment |
| --- | --- | --- |
| Qinghai Liu  (40) | Adhesive small bowel obstruction | Laparoscopic exploration; Biopsy of mesenteric nodes; Ileostomy |
| Jiunian Zheng  (12) | Adhesive incomplete small bowel obstruction | conservative |
| Guangli Huang  (5) | Acute Adhesive small bowel obstruction | Laparoscopic exploration; Intestinal adhesion release |
| Chuanguo Sun  (9) | Neoplastic incomplete small bowel obstruction | conservative |
| Jitu Wu  (68) | Acute Adhesive small bowel obstruction | conservative |
| Yinggeng Wang  (233) | Adhesive incomplete small bowel obstruction | conservative |
| Youxiong Lin  (17) | Adhesive small bowel obstruction | conservative |
| Jinmeng Zhang  (84) | Neoplastic small bowel obstruction | Cesarean section; Sigmoid colon perforation repair; Descending colonic tab stoma |
| Hao Cheng  (182) | Adhesive incomplete small bowel obstruction | Cesarean section; Intestinal adhesion release |
| Xunan Chen  (94) | Incomplete small bowel obstruction | conservative |
| Renqiao Hong  (37) | Acute mechanical small bowel obstruction | Laparoscopic exploration; Intestinal adhesion release |
| Mouyi Ye  (60) | Mesenteric torsion with subtotal small bowel necrosis;  Acute small bowel obstruction | Cesarean section; Intestinal adhesion release; Repositioning of intestinal torsion; Enterotomy and decompression; Subtotal colectomy |
| Liankai Xue  (44) | Acute adhesive incomplete small bowel obstruction | conservative |
| Xueshuang Yin  (9) | Torsional small bowel obstruction | Laparoscopic exploration; Intestinal torsion repositioning intermediate open abdomen abdominal adhesion release; Partial ileal resection |
| Longqiang Chen  (1) | Fecalithic complete small bowel obstruction | conservative |
| Zhenkang Huang  (10) | Acute adhesive incomplete small bowel obstruction | conservative |
| Jianxing Wu  (9) | Adhesive incomplete small bowel obstruction | conservative |

**Supplementary table 2: the discriminative effectiveness of LOS risk score**

| **Parameters** | **Non-SAE** | **SAE** | **p-value** |
| --- | --- | --- | --- |
| Score 0, n(%) | 58 (20.9) | 12 (4.3) | **＜0.001** |
| Score 1, n(%) | 205 (73.7) | 3 (1.1) |  |

LOS, length of stay; SAE, severe adverse even.
